# Clinical and Genetic Findings in Patients With Palmoplantar Keratoderma

**DOI:** 10.1001/jamadermatol.2024.4824

**Published:** 2024-12-04

**Authors:** Stine Bjørn Gram, Klaus Brusgaard, Ulrikke Lei, Mette Sommerlund, Gabrielle Randskov Vinding, Sondre Olai Kjellevold Sleire, Alex Hørby Christensen, Sanne Pedersen Fast, Rasmus Bach, Anette Bygum, Lilian Bomme Ousager

**Affiliations:** 1 Department of Clinical Genetics, Odense University Hospital, Odense, Denmark; 2 Department of Clinical Research, University of Southern Denmark, Odense, Denmark; 3 European Reference Network for Rare Skin Diseases, Odense and Roskilde, Denmark; 4 Department of Dermatology and Allergy, Copenhagen University Hospital, Herlev and Gentofte Hospital, Herlev, Denmark; 5 Department of Dermatology, Aarhus University Hospital, Aarhus, Denmark; 6 Department of Dermatology, Zealand University Hospital, University of Copenhagen, Roskilde, Denmark; 7 Department of Cardiology, Rigshospitalet and Herlev-Gentofte Hospitals, Hellerup, Denmark; 8 Fredericia Hudklinik, Fredericia, Denmark; 9 Department of Dermatology and Allergy Centre, Odense University Hospital, Odense, Denmark; 10 Hudklinikken Kolding, Kolding, Denmark

## Abstract

**Importance:**

Palmoplantar keratoderma poses diagnostic challenges due to its clinical and genetic heterogeneity, and knowledge on the value of systematic genetic testing on clinically well-described patient cohorts is sparse.

**Objective:**

To improve knowledge of the clinical and genetic spectrum of patients with palmoplantar keratoderma.

**Design, Setting, and Participants:**

This cohort study prospectively recruited patients and affected family members with palmoplantar keratoderma between September 1, 2016, and December 31, 2022. Patients were recruited from private practitioners in dermatology and dermatology departments in Denmark. Study participants were patients 18 years or older either newly diagnosed with palmoplantar keratoderma or being followed up for the disease at referral centers.

**Main Outcomes and Measures:**

Phenotypes and clinical subtypes were classified. Genetic testing was performed by whole-exome or genome sequencing using an in silico panel containing genes related to palmoplantar keratoderma, or by Sanger sequencing for specific variants. Descriptive analysis, such as proportions and frequency, were used to describe clinical characteristics, distribution of disease-causing variants, and genotype-phenotype associations.

**Results:**

This study included 142 study participants from 76 families (90 [63%] female; median [range] age, 52 [18-92] years). Clinical subtypes included 42 punctate (55%), 26 diffuse (34%), 5 focal (7%), and 3 striate (4%). A genetic diagnosis was found in 63 of 76 families (83%), including 27 disease-causing variants within 13 different genes: *AAGAB* (n = 39), *DSG1* (n = 8), *KRT1* (n = 3), *DSP* (n = 2), *KRT9* (n = 2), *AQP5* (n = 2), *KRT16* (n = 1), *SERPINA12* (n = 1), *ABCA12* (n = 1), *COL7A1* (n = 1), *CARD14* (n = 1), *DST* (n = 1), and *LORICRIN* (n = 1). All participants with *AAGAB* variants presented with punctate palmoplantar keratoderma, showing a clear genotype-phenotype correlation. The other subtypes (diffuse, focal, and striate) proved more challenging to clinically subclassify, and disease-causing variants were identified in 12 genes, contributing to more complex genotype-phenotype patterns. Patients with palmoplantar keratoderma due to *DSP* variants were found, which is important to identify because of an associated risk of cardiomyopathy.

**Conclusion and Relevance:**

This study provides novel insights into the clinical and genetic spectrum of patients with palmoplantar keratoderma. It demonstrates the value of genetic testing for accurate diagnoses and to distinguish between different subtypes. The established and well-described cohort lays the foundation for future research in palmoplantar keratoderma.

## Key Points

**Question:**

What is the clinical and genetic spectrum in a large cohort of Danish patients with palmoplantar keratoderma?

**Findings:**

In this cohort study, 142 patients with palmoplantar keratoderma from 76 families underwent deep phenotyping as well as genotyping, which yielded a diagnosis in 83% and identified 27 disease-causing variants across 13 genes: *AAGAB, DSG1, KRT1, DSP, KRT9, AQP5, LORICRIN, KRT16, SERPINA12, COL7A1, ABCA12, CARD14,* and *DST. AAGAB* variants correlated with punctate palmoplantar keratoderma, whereas other subtypes showed more complex genotype-phenotype patterns.

**Meaning:**

This study expands the phenotypic and genetic characteristics of palmoplantar keratoderma and highlights the importance of genetic testing in accurate diagnosis and subtype differentiation (eg, associated risks, such as cardiomyopathy).

## Teaser

This cohort study assesses the clinical and genetic spectrum of patients with palmoplantar keratoderma.

## Introduction

Hereditary palmoplantar keratoderma (PPK) is a group of rare skin disorders characterized by hyperkeratinization of palms and soles. The disease presents as hard and thickened skin and is often accompanied by pain, sweating, and an unpleasant odor. The disease is categorized into 4 clinical subtypes based on the pattern of hyperkeratosis: punctate, diffuse, focal, or striate.^1^ Additionally, PPK can be classified as (1) isolated PPK, affecting only the palms and soles; (2) part of more generalized skin diseases; or (3) part of syndromes, with increased risk of extracutaneous manifestations.^1,2^ Consequently, PPK is a heterogeneous disease and often challenging for clinicians to diagnose and manage.

Our understanding of the genetic basis of PPK has greatly improved in recent years but is primarily based on single cases, familial cases, and smaller case series. Only a few larger-scale cohort studies on specific subtypes have been reported,^3,4,5^ and the existing literature predominantly describes patients with specific phenotypes or genotypes. Apart from a previously published cohort of 64 Finnish patients by Harjama et al,^6^ only minimal literature covers unselected cohorts of patients as they present with different clinical subtypes in the dermatologic setting. Additional large, well-characterized cohorts of patients with PPK are necessary to assess the value of systematic genetic testing and to better understand the clinical and genetic spectrum of the disease.

The aim of our study was to examine the clinical and genetic characteristics of patients with PPK. To achieve this, we established a cohort of 142 patients from 76 families across Denmark to (1) describe the phenotypes of different clinical subtypes of PPK, (2) investigate the genetic causes and determine the diagnostic yield of systematic genetic testing, and (3) explore correlations between phenotypes and genotypes.

## Methods

### Participants

In this cohort study, participants were prospectively recruited in 2 steps: (1) from the Department of Dermatology and Allergy Centre, Odense University Hospital, Odense, Denmark, between September 1, 2016, and December 31, 2022, in collaboration with dermatology private practices in the Region of Southern Denmark; and (2) from other dermatology departments in Denmark invited to recruit patients between January 1, 2021, and December 31, 2022. Recruitment was performed by dermatologists (U.L., M.S., G.R.V., S.P.F., R.B., and A.B.) experienced in rare skin diseases. The inclusion criteria were newly diagnosed PPK or patients being followed up for the disease. Exclusion criteria were acquired PPK or age younger than 18 years. Affected family members were subsequently invited to join the study. Participants were categorized as probands, referring to the initial recruited patients, and relatives, representing included family members. Patients who agreed to participate in the study were invited to meet the principal investigator (S.B.G.) in person, by video consultation, or by telephone, depending on the patient’s preference (and/or COVID-19 recommendations at the time). The study was conducted in accordance with the Declaration of Helsinki^7^ and approved by the Regional Committees on Health Research Ethics for Southern Denmark and the Danish Data Protection Agency. Written informed consent was obtained from all participants. This study followed the Strengthening the Reporting of Observational Studies in Epidemiology (STROBE) reporting guideline.

### Phenotyping of Study Participants

The principal investigator (S.B.G.) interviewed each participant and collected information on age at onset; location (palms, soles, or both); progression of symptoms; aquagenic whitening; excessive sweating, odor, and fungal infections from hands and feet; woolly and/or curly hair; and present or previous treatment with systemic retinoids. Participants were asked about medical history, including past and present skin disease. Detailed pedigree and family history of PPK were obtained. Clinical examination of hands and feet, along with any other noticeable manifestations (eg, nails and hair), was performed in person (n = 54) or remotely using clinical images (n = 88). Clinical findings and subtype classification were discussed with at least 1 highly experienced dermatologist (A.B.) using clinical images of each participant.

Probands were categorized in clinical subtypes based on the pattern of hyperkeratosis: punctate (multiple papular hyperkeratotic lesions), diffuse (generalized hyperkeratosis covering most of the surface of palms and/or soles), focal (if localized hyperkeratotic lesions only on pressure points), or striate (if any signs of linear hyperkeratotic lesions). To avoid investigator bias related to the clinical or genetic classification of probands, we categorized relatives as having punctate or nonpunctate PPK.

Transgrediens, defined as lesions extending to the dorsal surfaces through continuous spread or lesions on areas such as knuckles or periungual skin, was recorded for participants with nonpunctate PPK. Different phenotypes are presented in eFigure 1 in Supplement 1.

### Genetic Testing

Three test strategies were used for the included probands: (1) 52 probands underwent testing with an in-house exome- or genome-based in silico panel designed by the authors, comprising 94 genes related to PPK (eAppendix in Supplement 1); (2) 14 probands underwent focused analysis of *AAGAB* (OMIM 614888) c.370C>T using bidirectional Sanger sequencing based on a clinical diagnosis of punctate PPK (because a common occurrence of this variant was found in this subtype during the study); and (3) 10 probands had previously undergone genetic testing at other laboratories.

Relatives were tested by bidirectional Sanger sequencing for the identified variants in the proband of each family or tested using the above-mentioned in silico panel. The eAppendix in Supplement 1 details the sequencing methods.

Identified variants were interpreted based on type (missense, nonsense, frameshift, splice site, or deletion/duplication), use of software predictors included in the Alamut Visual Plus Software (SOPHiA GENETICS) and Combined Annotation Dependent Depletion score,^8^ allele frequencies in the Genome Aggregation Database, previous registration in Human Gene Mutation Database^9^ and/or ClinVar,^10^ as well as the application of the American College of Medical Genetics and Genomics criteria.^11^ Segregation analysis was performed when possible.

### Statistical Analysis

Data were analyzed using descriptive analysis. Clinical characteristics of genetic subtypes were described and presented in tables and diagrams. The study data were collected, managed, and stored using REDCap (Research Electronic Data Capture; Vanderbilt University) tools hosted at Odense University Hospital.^12,13^

## Results

### Study Population

 We prospectively enrolled 76 probands and 66 affected relatives with PPK, resulting in a total study population of 142 participants (90 [63%] female and 52 [37%] male; median [range] age, 52 [18-92] years). Demographics and clinical characteristics of the probands are summarized in Table 1.

**Table 1.** Demographics and Clinical Presentation of 76 Probands With PPK

Variable	No. (%) of patients^a^				
	Punctate (n = 42)	Diffuse (n = 26)	Focal (n = 5)	Striate (n = 3)	Total (N = 76)
Age at inclusion, median (range), y	58 (32-82)	42 (19-75)	27 (18-70)	28 (25-35)	52 (18-92)
Sex					
Male	14	10	1	2	27
Female	28	16	4	1	49
Recruited from					
Region of Southern Denmark^b^	31	18	2	2	53
Other regions in Denmark^c^	11	8	3	1	23
Age at onset, y					
Median (range)	19.0 (5.0-47.0)	5.3 (0-44.0)	15.0 (4.0-21.0)	6.0 (4.5-6.5)	14.5 (0-47.0)
Age group					
Birth	0	3 (12)	0	0	3 (4)
<1	0	4 (15)	0	0	4 (5)
1-9	3 (7)	8 (31)	1 (20)	3 (100)	15 (20)
10-19	17 (41)	5 (19)	2 (40)	0	24 (32)
>19	19 (45)	4 (15)	1 (20)	0	24 (32)
Other	3 (7)	2 (8)	1 (20)	0	6 (8)
Palms and/or soles					
Palms and soles	40 (95)	24 (92)	3 (60)	3 (100)	70 (92)
Palms only	0	0	0	0	0
Soles only	2 (5)	2 (8)	2 (40)	0	6 (8)
Changes of PPK with age					
Stable	8 (19)	12 (46)	2 (40)	1 (33)	23 (30)
Progression	31 (74)	6 (23)	3 (60)	1 (33)	41 (54)
Improvement	1 (2)	3 (12)	0	1 (33)	5 (7)
Other	2 (5)	3 (12)	0	0	5 (7)
NA		2 (8)	0	0	2 (3)
Transgrediens, No./total No. (%)	NR	14/21 (71)	0/5 (0)	2/3 (67)	16/29 (55)
Associated symptoms, No./total No. (%)					
Pain or soreness	29/42 (69)	16/26 (62)	4/5 (80)	3/3 (100)	52/76 (68)
Sweat	14/40 (35)	11/25 (44)	1/3 (33)	0/3 (0)	26/71 (37)
Odor	15/41 (37)	13/26 (50)	3/5 (60)	3/3 (100)	34/75 (45)
Aquagenic whitening	34/40 (85)	16/25 (64)	1/4 (25)	2/2 (100)	53/71 (75)
History of fungal infections	4/40 (10)	7/23 (30)	4/5 (80)	2/3 (67)	17/71 (24)
Woolly or curly hair	1/40 (3)	2/24 (8)	1/5 (20)	2/3 (67)	6/72 (8)
Treatment with systemic retinoids (present or previously), No./total No. (%)	20/40 (50)	8/25 (32)	1/5 (20)	2/3 (67)	31/73 (42)
Self-reported effect	13/18 (72)	5/6 (83)	0/1 (0)	1/2 (50)	20/27 (74)

Abbreviations: NA, not applicable; NR, not registered; PPK, palmoplantar keratoderma.

^a^ Unless otherwise indicated.

^b^ Recruited from the Department of Dermatology and Allergy, Odense University Hospital, and though collaborations with dermatology private practices in the Region of Southern Denmark.

^c^ Recruited from hospital settings.

### Phenotypic Presentation of Probands

Punctate PPK was the most prevalent subtype (42 of 76 [55%]), followed by diffuse (26 of 76 [34%]), focal (5 of 76 [7%]), and striate (3 of 76 [4%]). Age at onset ranged from 0 to 47.0 years across all clinical subtypes. Patients with punctate PPK reported the highest median age at onset of 19.0 years (range, 5.0-47.0 years), whereas those with diffuse PPK had the earliest median age at onset of 5.3 years (range, 0-44.0 years). Most patients were affected on both palms and soles (70 of 76 [92%]). Transgrediens was noted in approximately half of probands with nonpunctate PPK, predominantly among diffuse PPK. Among all clinical subtypes of PPK, most probands had experienced either worsening of symptoms (41 of 76 [54%]) or stable disease with age (23 of 76 [30%]), whereas only a few (5 of 76 [7%]) reported improvement. Most probands reported pain or soreness associated with PPK (52 of 76 [68%]). A substantial proportion also reported excessive sweating (26 of 71 [37%]), malodor (34 of 75 [45%]), and current or previous history of fungal infections (17 of 71 [24%]). Aquagenic whitening was reported by 53 of 71 (75%), and a 6 of 72 patients (8%) exhibited curly hair. A total of 31 probands had a history of present or previous treatment with systematic retinoids, whereas this applied to only 5 relatives.

### Genetic Characterization

#### Family History of PPK

Most probands (63 of 72 [88%]) reported affected family members, and all but 1 (61 of 62 [98%]) had affected first-degree relatives. Selected pedigrees are presented in eFigure 2 in Supplement 1.

#### Genetic Variants Detected in Probands

**Figure 1.**
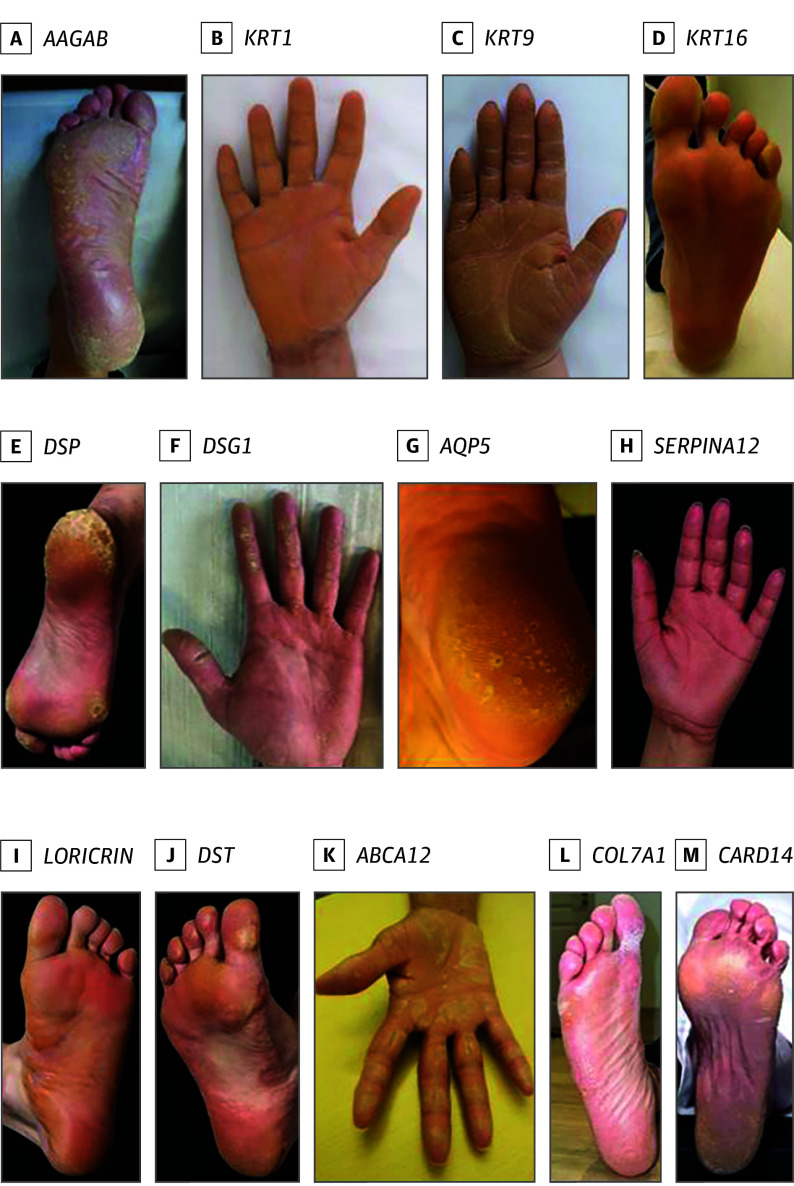
Clinical Images of Patients With Palmoplantar Keratoderma Clinical images illustrating phenotypic features of the genotypes identified in the cohort: *AAGAB* (proband 70), *KRT1* (proband 32), *KRT* 9 (proband 30), *KRT16* (proband 13), *DSP* (proband 60), *DSG1* (proband 41), *AQP5* (relative 55), *SERPINA12* (proband 64), *LORICRIN* (proband 4), *DST* (proband 59), *ABCA12* (proband 19), *COL7A1* (proband 63), and *CARD14* (proband 31).

A molecular genetic diagnosis was identified in 63 of 76 probands. The variants were found in 13 different genes and distributed as follows: *AAGAB* (OMIM 614888) (n = 39 of 76), *DSG1* (OMIM 125670) (n = 8 of 76), *KRT1* (OMIM 139350) (n = 3 of 76), *DSP* (OMIM 125647) (n = 2 of 76), *KRT9* (OMIM 607606) (n = 2 of 76), *AQP5* (OMIM 600442) (n = 2 of 76), *KRT16* (OMIM 148067) (n = 1 of 76), *SERPINA12* (OMIM 617471) (n = 1 of 76), *ABCA12* (OMIM 607800) (n = 1 of 76), *COL7A1* (OMIM 120120) (n = 1 of 72), *CARD14* (OMIM 607211) (n = 1 of 76), *DST* (OMIM 113810) (n = 1 of 76), and *LORICRIN* (OMIM 152445) (n = 1 of 76). A total of 27 pathogenic (n = 14) or likely pathogenic (n = 13) variants were identified; 13 of these variants were novel in PPK. Most common was the recurrent variant *AAGAB*, c.370C>T, p.(Arg124Ter) detected in 35 probands. Identical variants were also identified in 2 probands with the variants: *AAGAB* (chr15:67534480_67579632del), *KRT9* (c.487C>T), and *AQP5* (c.562C>T). Nine of 13 genes had autosomal dominant inheritance pattern with study participants being heterozygous, whereas 4 genes were linked to autosomal recessive inheritance. For a detailed summary of detected variants and additional clinical descriptions, see Table 2, Figure 1, and eTables 1 to 4 in Supplement 1.^14,15,16,17,18,19,20,21,22,23,24,25,26,27,29,30^

**Table 2.** Genetic Variants Identified in a Danish Cohort of 76 Probands With PPK

Gene	Proband identification	Type of variant	Nucleotide change	Amino acid change	Interpretation	Novelty
**Isolated PPK**						
*AAGAB*	35 Cases^a^	Nonsense	c.370C>T	p.(Arg124Ter)	P	Giehl et al^14^^b^
	P34	Initiator codon	c.2T>A	p.Met1?	P	Giehl et al^15^
	P42 and P52	Deletion	Promotor + exon 1^c^	p.?	LP	Novel^d^
	P58	Deletion	Exon 1^e^	p.?	LP	Novel^d^
*DSG1*	P2	Frameshift	c.1421delC	p.(Thr474Ilefs*88)	LP	Novel
	P23	Splice site	c.1005 + 1G>T	p.?	P	Novel
	P26	Nonsense	c.2659C>T	p.(Arg887Ter)	P	Has et al^16^
	P41	Nonsense	c.76C>T	p.(Arg26Ter)	P	Hunt et al^17^
	P45	Nonsense	c.1199C>G	p.(Ser400Ter)	P	Novel
	P48	Deletion	c.(372_373)_(1005 + 1006)del	p.?	LP	Novel
	P50	Frameshift	c.1947_1950delGAGA	p.(Arg650Ter)	LP	Novel
	P68	Splice site	c.1265 + 1G>A	p.?	LP	Novel
*KRT9*	P30 and P43	Missense	c.487C>T	p.(Arg163Trp)	P	Reis et al^18^
*AQP5*	P46 and P71	Missense	c.562C>T	p.(Arg188Cys)	P	Blaydon et al^19^
*SERPINA12*	P64	Frameshift	c.594delC	p.Gly199Alafs*3	LP	Novel
		Frameshift	c.594delC	p.Gly199Alafs*3	LP	
*KRT16*	P13	Missense	c.379C>T	p.(Arg127Cys)	P	Shamsher et al^20^
*KRT1*	P73	Missense	c.608A>G	p.(Gln203Arg)	LP	Novel
**PPK as part of other genodermatoses**						
*KRT1*	P32	Missense	c.1424T>C	p.(Leu475Pro)	LP	Lee et al^21^ (participant previously described^22^)
	P44	In-frame deletion	c.673_702del30	p.His225_Phe234del	LP	Participant previously described^22^
*LORICRIN*	P4	Frameshift	c.792dupC	p.(Ile265Hisfs*71)	P	Novel
*CARD14*	P31	Missense	c.412G>A	p.(Glu138Lys)	LP	Participant previously described^23^
*DST*	P59	Frameshift	c.7544dupA/	p.(Gln2516Alafs*6)/	P	Novel
		Frameshift	c.7544dupA/	p.(Gln2516Alafs*6)		
*COL7A1*	P63	Nonsense	c.5797C>T/	p.(Arg1933Ter)/	P	Whittock et al^24^
		Nonsense	c.8584G>T	p.(Glu2862Ter)	P	Chen et al^25^
*ABCA12*	P19	Deletion-insertion	c.1002_1004delAACinsT/	p.(Thr335Alafs*5)/	LP	Participant previously described^26^
		Missense	c.6263T>C	p.(Leu2088Pro)	VUS^f^	
**PPK with risk of associated diseases**						
*DSP*	P21	Nonsense	c.2821C>T	p.(Arg941Ter)	P	Novel (PPK)^g^
	P60	Frameshift	c.175dupA	p.(Thr59Asnfs*34)	LP	Participant previously described^27^

Abbreviations: LP, likely pathogenic; P, pathogenic; PPK, palmoplantar keratoderma; VUS, variant of uncertain significance.

^a^ Patients 3, 5, 8 to 12, 15, 18, 20, 22, 24, 25, 28, 29, 33, 35 to 39, 47, 49, 51, 53 to 56, 61, 65 to 67, 70, 72, and 75.

^b^ Twenty of the study participants have previously been described.^28^

^c^ (chr15:67534480_67579632)del.

^d^ Other deletions, including exon 1 of *AAGAB,* have been reported in ClinVar and Harjama et al^6^ and Pöhler et al^29^; however, the exact genomic coordinates have not been reported.

^e^ (c.1-6607)_(c.74-95)del.

^f^ Classified as VUS according to the American College of Medical Genetics and Genomics guidelines.^11^ However, supporting evidence that the variant affects the protein structure can be found in eFigure 3 in Supplement 1.

^g^ The variant has previously been reported in relation to cardiomyopathy^30^ but is novel in relation to PPK.

#### Diagnostic Yield and Molecular Genetic Diagnoses

**Figure 2.**
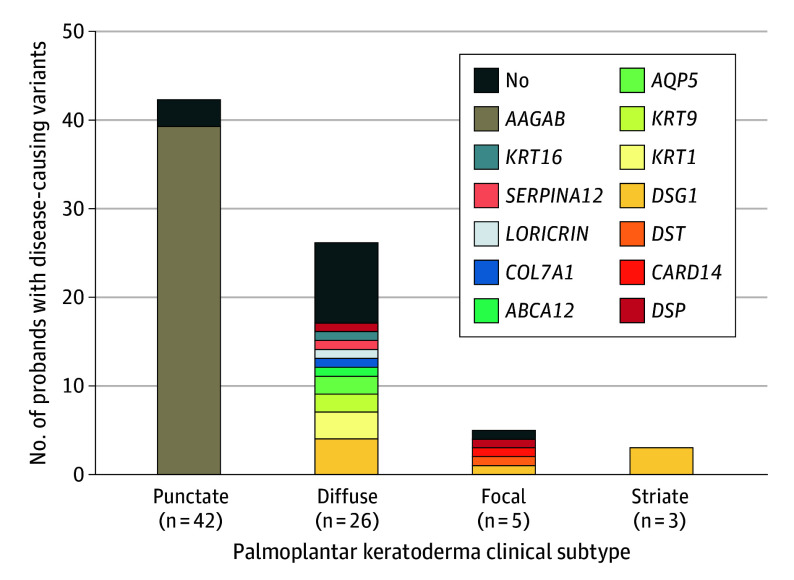
Clinical Subtypes and Molecular Genetic Diagnosis in 76 Families With Palmoplantar Keratoderma Distribution of clinical subtypes based on patterns of hyperkeratosis and overview of genes with disease-causing variants identified in each clinical subtypes.

A genetic diagnosis was identified in 63 of 76 probands, corresponding to a diagnostic yield of 83%. The highest rates were among striate (3 of 3 [100%]) and punctate PPK (39 of 42 [93%]), followed by diffuse (17 of 26 [65%]) and focal (4 of 5 [80%]) (Figure 2).

Testing of affected relatives identified a disease-causing variant in 54 additional study participants (Figure 2; eTable 2 in Supplement 1), whereas 12 affected relatives were ineligible for genetic testing because no disease-causing variants were identified in the probands in these families. Adding the number of affected relatives with disease-causing variants to the 63 probands with a genetic diagnosis, a total of 117 study participants (82% of the entire study population) received a molecular genetic diagnosis of PPK.

#### Genetic Subtypes and Phenotypic Features

**Figure 3.**
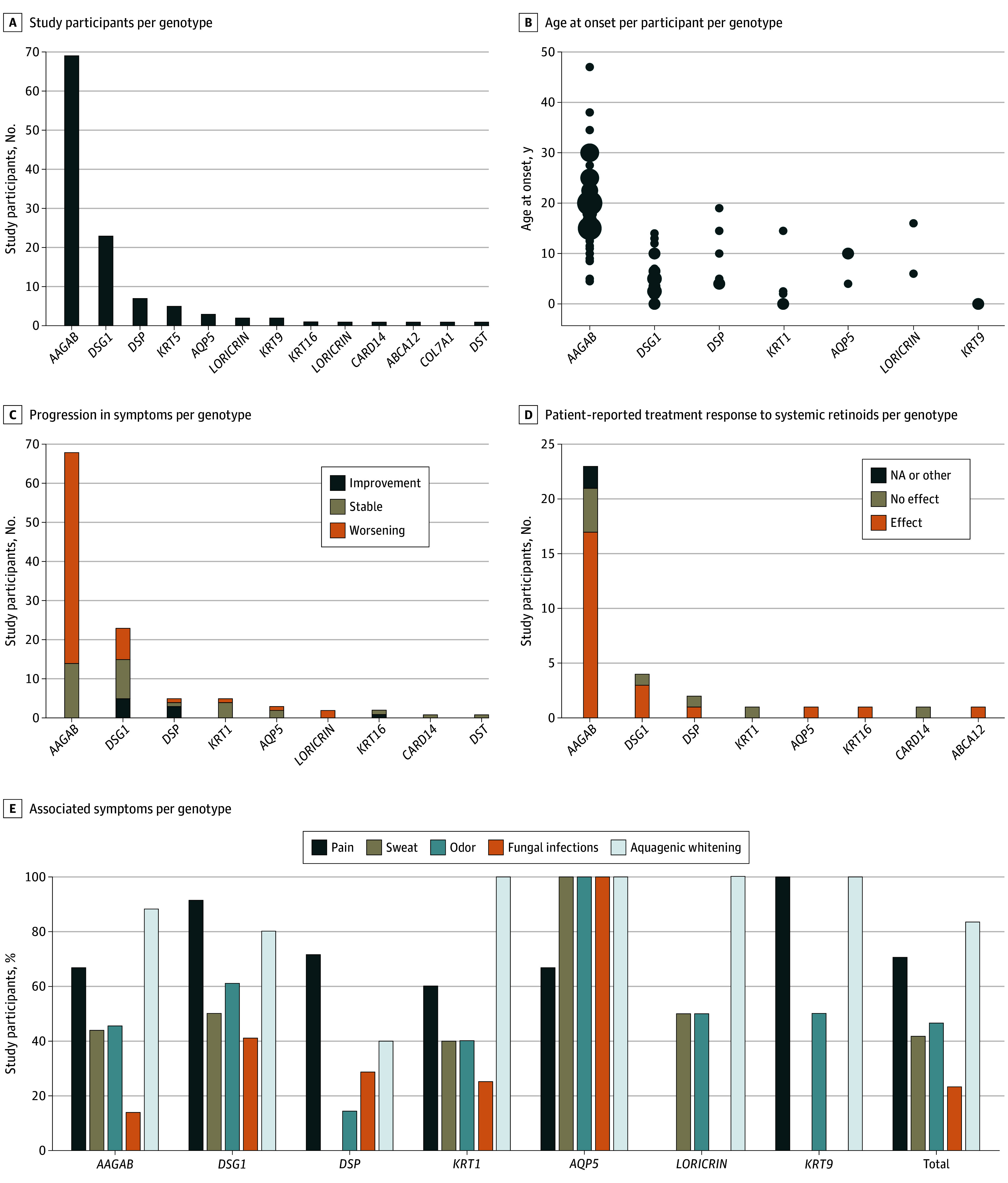
Genetic Subtypes and Phenotypic Features A, Number of study participants per genotype. B, Age at onset of palmoplantar keratoderma for each participant. Enlarged dots represent more participants with the same age at onset. C, Progression in symptoms per genotype. D, Patient-reported treatment response to systemic retinoids per genotype. E, Associated symptoms per genotype. Total indicates the total number of participants with a genetically confirmed diagnosis. Numbers presented are based on informative cases (cases with missing data are omitted). For panels B and E, clinical data per genotype are reported for genes represented by at least 2 participants in the study cohort. Data and specific numbers and percentages are also available in eTable 2 in Supplement 1. NA indicates not applicable.

On the basis of clinical and genetic data, we evaluated genotype-phenotype correlation in our cohort of 117 participants (probands and relatives) with molecular genetic-confirmed PPK. Demographic data and clinical features related to each genotype are detailed in eTable 2 in Supplement 1. *AAGAB* was the most frequent genotype (n = 69), followed by *DSG1* (n = 23), *DSP* (n = 7), *KRT1* (n = 5), *AQP5* (n = 3), *LORICRIN* (n = 2), *KRT9* (n = 2), and single cases of *KRT16*, *SERPINA12, CARD14*, *ABCA12*, *DST*, and *COL7A1*. All participants with *AAGAB* had punctate PPK, which was also the only genetic diagnosis found within this group. All 12 other genotypes were identified in participants with nonpunctate PPK (Figure 3A). Participants with *AAGAB* variants had a median age at onset of 18.0 years (range, 4.5-47.0 years), whereas other genotypes represented in the cohort with 2 or more individuals ranged from a median age at onset of 0 to 11.0 years (Figure 3B). Participants with *AAGAB* variants mostly reported worsening symptoms with age (54 of 69 [78%]). Participants with *DSG1* variants reported various disease courses, with stable disease being the most common (10 of 23 [43%]), whereas participants with *DSP* variants, although small in number, mostly reported improvement of symptoms (3 of 5 [60%]) (Figure 3C). None of the participants with *DSP* variants presented with woolly hair as a diagnostic clue of a cardiocutaneous syndrome. The proband in 1 of the 2 families had prominent curly hair, as did the affected relatives. The proband in the other family did not share this characteristic. Other associated symptoms, such as pain or soreness (82 of 117 [70%]), excessive sweating (46 of 109 [42%]), odor (54 of 114 [47%]), and fungal infections (25 of 110 [23%]) were frequently reported in the entire cohort with genetic confirmed PPK (eTable 2 in Supplement 1; Figure 3D).

## Discussion

We collected phenotypic and genetic data on a large cohort of patients with PPK, comprising 142 patients from 76 families in Denmark. The genetic cause of disease was identified in 63 families corresponding to 117 study participants. Punctate PPK was the most prevalent clinical subtype, with *AAGAB* as the single disease-causing gene identified in most cases. In participants with nonpunctate PPK (diffuse, focal, or striate), we identified disease-causing variants in 12 different genes (*DSG1, DSP, KRT1, KRT9, AQP5, LORICRIN, KRT16, SERPINA12, DST, COL7A1, CARD14,* and *ABCA12*). Among these, 2 families were diagnosed with variants in *DSP*, a gene known to be related to cardiomyopathy as well as PPK.

The clinical characteristics of cases with *DSP* variants did not clearly distinguish themselves from the other nonpunctate cases, making it challenging to identify them solely on clinical features. This finding highlights the necessity of genetic testing to differentiate specific subtypes, especially for identifying cases where cardiac examination and surveillance are crucial.

### Diagnostic Yield of Genetic Testing in Patients With PPK

Our systematic genetic evaluation of patients with PPK resulted in a remarkable diagnostic yield of 83%. This high rate may potentially have been influenced by patients with affected family members being more likely to participate in the study and clinicians being more attentive to recruiting such patients. However, the results support the hereditary nature of the disease. The only comparable study, which was performed on 64 patients with different clinical subtypes of PPK in Finland,^6^ reported a genetic diagnosis in 48%. The differences in diagnostic yield likely represent different distributions of clinical subtypes and the presence of different founder variants in the Danish and Finnish populations. In the Finnish cohort, 30% had focal PPK,^6^ and no disease-causing variants were identified in this subgroup. In our study, this clinical subtype only represented 7%, and we found a genetic diagnosis in 80% of our cases. Punctate PPK constituted the most common subtype in our study, with a very high diagnostic yield of 93%, mainly explained by identification of a specific variant in *AAGAB*, c.370C>T, p.(Arg124Ter). We have shown that this is a founder variant in 20 of the study participants originally included from the Region of Southern Denmark^28^ but also identified in study participants from other parts of Denmark. This clinical subtype also had the highest diagnostic yield in the Finnish cohort (69%). A genetic diagnosis was found in a comparable percentage in patients with diffuse PPK, despite the presence of founder variants in *SERPINB7* and *AQP5* in the Finnish patients.

In 13 of 76 probands, we were unable to identify a genetic diagnosis. Most of these probands (n = 11) reported affected first-degree family members and had pedigrees highly indicative of monogenic disease. Furthermore, a number of families shared interesting phenotypic similarities (eg, transgredient, erythematous PPK following an autosomal dominant inheritance pattern) (eTable 5 in Supplement 1). Therefore, new genes related to PPK most likely remain to discovered.

### Punctate vs Nonpunctate PPK

Punctate PPK was the most observed clinical subtype in our cohort. The variant *AAGAB*, c.370C>T accounted for most cases (35 of 42 [83%]), which turned out to be a founder variant.^28^ Overall, variants in *AAGAB* were the single genetic cause identified in punctate PPK, consistent with existing knowledge about the molecular genetic cause of isolated punctate PPK.^14,15^ In addition, the total absence of *AAGAB* in nonpunctate PPK underlines a rather simple association between phenotype and genotype in isolated punctate PPK. This subtype has long been suggested to be associated with an increased risk of malignant neoplasms. However, a recent systematic review of the literature concluded that the quality of studies supporting this association is too poor to confirm it.^31^

Nonpunctate PPK showed greater clinical and genetic heterogeneity. Classic clinical subtypes were present, including several clearly focal cases and, for example, pronounced diffuse keratoderma linked to *KRT9.* The nonpunctate group also included cases in which clinical subclassification (diffuse, focal, or striate) proved challenging and led to discussions among the investigators. This finding applied to several patients in our cohort with *DSG1* variants, a condition associated with varying patterns of hyperkeratinization.^32^ Still, in several other cases (see comments below eTable 1 in Supplement 1), distinguishing between especially mild diffuse and focal subtypes was challenging. Consequently, on the basis of our cohort, we suggest the broader term *nonpunctate* as an alternative, when clear signs of diffuse, focal, or striate subtypes are not evident.

Within nonpunctate cases, we identified disease-causing variants in 6 genes related to isolated PPK (*DSG1, KRT9, KRT1, AQP5, KRT16, *and* SERPINA12*) and 6 genes related to PPK and other skin symptoms (*KRT1, LORICRIN, DST, CARD14, COL7A1, *and* ABCA12*). Although the causal association of *COL7A1* to dystrophic epidermolysis bullosa is well established, its role in PPK requires further studies and validation. Likewise, variants in *ABCA12* are known to cause ichthyosis, with PPK also reported in a few patients.^33,34^ Furthermore, 1 gene associated with syndromic PPK (*DSP*) was identified.

This large genetic heterogeneity combined with clinical similarities within the nonpunctate group emphasizes the relevance of genetic testing for subdiagnosis. The results also highlight the important aspect of genetic testing to identify patients at risk for associated conditions as variants in *DSP*, a gene that, in addition to PPK, has been linked to cardiomyopathy, were identified in 2 included families.^35,36^ The 2 probands presented with diffuse and focal PPK, respectively. One of these had curly hair; the other did not. Therefore, no distinct and common clinical phenotype was seen, despite both probands being diagnosed with *DSP*-related disease. This observation highlights the paramount importance of genetic testing. The 2 *DSP* variants were found in 6% of the 34 nonpunctate cases, increasing to 7% (n = 2 of 29) after excluding 5 patients with more widespread dermatological symptoms (*KRT1*, *LORICRIN, ABCA12, COL7A1, *and* CARD14*). In our cohort, this corresponds to approximately 1 in 14 patients with nonpunctate PPK, emphasizing the role of genetic analysis in identifying patients requiring further cardiac investigations and surveillance. In the 2 *DSP* families in our cohort, family members were referred to cardiac investigations. None were diagnosed with *DSP*-related cardiomyopathy at the time of evaluation; however, in some of the cases, borderline findings were recorded (eTable 1 in Supplement 1). Accurate genetic diagnoses also has clinical value and is becoming increasingly relevant as new treatments are being developed for specific subtypes (eg, patients with pachyonychia congenita,^37^ 1 of whom was diagnosed in our cohort).

Regarding treatment, we also recorded that 36 study participants were currently or previously treated with systemic retinoids. Most reported positive effects from the treatment; however, systemic retinoids are known to cause various potential adverse effects, especially as long-term therapy, which often cause patients to discontinue the treatment despite symptom improvement. Therefore, the continued development of new treatments is extremely important, and well-established cohorts can serve as an important basis for trials.

### Strengths and Limitations

Our study has several strengths. The cohort size represents, to our knowledge, the largest study on a diverse clinical group of patients with PPK. Our prospective design ensured systematic, comprehensive data collection and minimizing of missing data compared with retrospective methods. Furthermore, all clinical (S.B.G., A.B.) and genetic (S.B.G., K.B.) data were assessed by the same 2 investigators, providing a high level of consistency.

Our study also has possible limitations. Our cohort may not fully represent all patients with PPK in Denmark because recruitment was not uniform across regions. Furthermore, patients with more severe disease may have been more prone to seek medical advice. All participants were recruited in person by clinicians with experience in rare skin diseases. However, due to inclusion during the COVID-19 pandemic, some clinical variables were assessed only by clinical images, potentially affecting accuracy. Despite our gene panel being comprehensive, the gene panel approach might have missed molecular diagnoses in genes not included in the panel. Furthermore, there are limitations related to the used sequencing techniques (eg, inability to detect variants in noncoding regions or risk of poor coverage of specific genes or exons). However, our study shows that testing with a customized gene panel is a rational approach for diagnosing most patients suspected of having hereditary PPK.

## Conclusions

This study provides valuable information on the clinical and genetic landscape of a large Danish cohort with PPK and shows the phenotypic and genetic diversity of the disease. A genetic diagnosis was identified in 83%, demonstrating the highly hereditary nature of the disease. The results also contribute to broadening the known variant spectrum, as several novel variants were identified. Punctate PPK was the most prevalent clinical subtype, solely caused by variants in *AAGAB*. Nonpunctate PPK was caused by variants in 12 different genes, including variants in *DSP*, a gene also linked to cardiomyopathy. Identification of cases with increased risk of cardiomyopathy is crucial, as sudden life-threatening cardiac events may be the first manifestation of disease. Because no distinct clinical features can differentiate these patients from others with nonpunctate PPK, genetic testing is essential for identifying patients in whom cardiac examinations and surveillance are crucial. This study illustrates the value of genetic testing in distinguishing between specific subtypes and ensuring accurate diagnosis.

## Notes

Supplement 1. **eAppendix. **Detailed Description of Methods, Genes Included in the *In-Silico* Gene Panel, and List of Other Laboratories Performing the Genetic Tests in 10 of the Included Patients **eFigure 1. **Examples of Different Clinical Features in Study Subjects With Palmoplantar Keratoderma **eFigure 2. **Selected Pedigrees of Families Included in the Study **eFigure 3. **Supporting Evidence That the Variant *ABCA12*, C.6263T>C, P.(Leu2088Pro) Affects Protein Function **eFigure 4.** Suggested Genetic Test Strategy Based on Clinical Subtype **eTable 1.** Clinical Findings and Family Data on Different Genotypes Identified in a Cohort With Palmoplantar Keratoderma **eTable 2. **Demographics and Clinical Presentation of 115 Study Subjects (Probands and Relatives) With Molecular Genetic Confirmed Palmoplantar Keratoderma **eTable 3. **Supplementary Information on the Genetic Variants Identified in the Cohort Including Information on Reference Sequences, Zygosity, gnomAD, Software, and ACMG Criteria and Interpretation **eTable 4. **Variants of Unknown Significance Identified in the 76 Probands With PPK **eTable 5.** Clinical Characteristics of the 13 Study Participants Without a Genetic Diagnosis

Supplement 2.
Data Sharing Statement
