# Supplementary material for: Clinical and Genetic Findings in Patients With Palmoplantar Keratoderma
Source: JAMA Dermatol. 2024 Dec 4;161(2):157–66. doi: 10.1001/jamadermatol.2024.4824 (PMC11618570; doi:10.1001/jamadermatol.2024.4824)
Supplement: Supplement 1. — eAppendix. Detailed Description of Methods, Genes Included in the In-Silico Gene Panel, and List of Other Laboratories Performing the Genetic Tests in 10 of the Included Patients eFigure 1. Examples of Different Clinical Features in Study Subjects With Palmoplantar Keratoderma eFigure 2. Selected Pedigrees of Families Included in the Study eFigure 3. Supporting Evidence That the Variant ABCA12, C.6263T>C, P.(Leu2088Pro) Affects Protein Function eFigure 4. Suggested Genetic Test Strategy Based on Clinical Subtype eTable 1. Clinical Findings and Family Data on Different Genotypes Identified in a Cohort With Palmoplantar Keratoderma eTable 2. Demographics and Clinical Presentation of 115 Study Subjects (Probands and Relatives) With Molecular Genetic Confirmed Palmoplantar Keratoderma eTable 3. Supplementary Information on the Genetic Variants Identified in the Cohort Including Information on Reference Sequences, Zygosity, gnomAD, Software, and ACMG Criteria and Interpretation eTable 4. Variants of Unknown Significance Identified in the 76 Probands With PPK eTable 5. Clinical Characteristics of the 13 Study Participants Without a Genetic Diagnosis [file jamadermatol-e244824-s001.pdf]

Supplementary Online Content

Gram SB, Brusgaard K, Lei U, et al. Clinical and genetic findings in patients with palmoplantar keratoderma. *JAMA Dermatol.* Published online December 4, 2024. doi:10.1001/jamadermatol.2024.4824

eAppendix. Detailed Description of Methods, Genes Included in the *In-Silico* Gene Panel, and List of Other Laboratories Performing the Genetic Tests in 10 of the Included Patients

eFigure 1. Examples of Different Clinical Features in Study Subjects With Palmoplantar Keratoderma

eFigure 2. Selected Pedigrees of Families Included in the Study

eFigure 3. Supporting Evidence That the Variant *ABCA12*, C.6263T>C, P.(Leu2088Pro) Affects Protein Function

eFigure 4. Suggested Genetic Test Strategy Based on Clinical Subtype

eTable 1. Clinical Findings and Family Data on Different Genotypes Identified in a Cohort With Palmoplantar Keratoderma

eTable 2. Demographics and Clinical Presentation of 115 Study Subjects (Probands and Relatives) With Molecular Genetic Confirmed Palmoplantar Keratoderma

eTable 3. Supplementary Information on the Genetic Variants Identified in the Cohort Including Information on Reference Sequences, Zygosity, gnomAD, Software, and ACMG Criteria and Interpretation

eTable 4. Variants of Unknown Significance Identified in the 76 Probands With PPK

eTable 5. Clinical Characteristics of the 13 Study Participants Without a Genetic Diagnosis

This supplementary material has been provided by the authors to give readers additional information about their work.

© 2024 Gram SB et al, *JAMA Dermatology*.

eAppendix. Detailed Description of Methods, Genes Included in the *In-Silico* Gene Panel, and List of Other Laboratories Performing the Genetic Tests in 10 of the Included Patients

Whole-exome-sequencing (WES) and whole-genome-sequencing (WGS)

WES was performed on blood samples received before 2020 and WGS was performed on blood samples received after 2020. Prior to sequencing, DNA was extracted from blood samples using a Freedom EVO DNA purification robot (Tecan) according to the manufacturers' protocols.

Whole-exome-based analysis: An exome DNA library was produced using SeqCapEZ Human Exome Kit v3.0 (Roche Ltd.). Sequencing was performed using NextSeq or NovaSeq 6000 (Illumina Inc.). An average of 95 % of the targeted exome bases was covered to a depth of 20X or greater. Downstream data analysis was performed using GenomeStudio TM Data Analysis Software (Illumina Inc. San Diego USA). Localisation and annotation of the genetic variants was done using GATK (Broad Institute) and ANOVAR.

Whole-genome-based analysis. A genome DNA library was produced using DNA PCR-Free Genome Prep. (Illumina) according to the manufacturers' protocols. Sequencing was performed using NovaSeq 6000 (Illumina Inc.). An average of 95% of the targeted exome bases was covered to a depth of 20X or greater. Downstream data analysis was done using DRAGEN software (Illumina).

The final data filtering was performed using VarSeq software (Golden Helix). Variants were filtered based on allele frequency < 0.03 and less than 5 homozygotes (Gnomad 2.1.0) and variant allele frequency (VAF) in reads $\geq 25\%$. We first looked for single-nucleotide-polymorphisms (SNP). If no likely pathogenic or pathogenic variants were identified, we subsequently evaluated copy-number-variants. Only variants within genes in the following gene panel were evaluated.

***In-silico* gene panel, palmoplantar keratoderma**

AAAS, AAGAB, ABCA12, ADAM10, ALOX12B, ALOXE3, ATP2A2, AQP5, BRAF, CARD14, CAST, CDH12, CERS3, COG6, COL14A1, COL20A1, CSTA, CTSC, CYP4F22, DSC2, DSG1, DSP, EDA1, EDAR, EDARADD, ENPP1, FAM83G, FECH, FERMT1, FLG2, GJA1, GJB2, GJB3, GJB4, GJB6, GRHL2, HPGD, HRAS, JUP, KANK2, KDSR, KLHL24, KRAS, KRT1, KRT5, KRT6A, KRT6B, KRT6C, KRT9, KRT10, KRT14, KRT16, KRT17, KRT83,

© 2024 Gram SB et al, *JAMA Dermatology*.

LIPN, LOR, MAP2K1, MAP2K2, MBTPS2, MTTS1, NECTIN4, NIPAL, NIPAL4, NLRP1, NTRK1, PERP, PKP1, PLEC, PIGO, PNPLA1, POMP, PTEN, PTPN11, RAF1, RSPO1, RHBDF2, SASH1, SDR9C7, SERPINB7, SERPINB8, SLCO2A1, SLURP1, SMARCAD1, SNAP29, SRD5A3, ST14,STS, TAT, TGM1, TP63, TRPV3, USB1, VPS33B, WNT10A. Updated in June 2024 where unsolved cases were tested with the following added genes: AP1B1, AP1S1, CTSZ, KLF4, LSS, TUFT1, SERPINA12

List of other laboratories performing the genetic testing of listed study participants

P43	*KRT9*<br>c.487C>T, p.(Arg163Trp)	Department of Clinical Genetics, Aarhus University Hospital	Sanger sequencing of KRT9
P56	*AAGAB*<br>c.370C>T, p.Arg124*	Department of Clinical Genetics, Aarhus University Hospital	Exome-sequencing-based *in-silico* panel containing 28 genes related to palmoplantar keratoderma
P58	*AAGAB*<br>Heterozygous deletion of exon 1	Department of Clinical Genetics, Rigshospitalet, Copenhagen, Denmark	Exome-sequencing-based *in-silico* panel containing 353 genes related to dermatological diseases.
P59	*DST*<br>c.7544dupA, p.(Gln2516Alafs*6))	Department of Clinical Genetics, Rigshospitalet, Copenhagen, Denmark	Exome-sequencing-based *in-silico* panel containing 352 genes related to dermatological diseases.
P63	*COL7A1*<br>c.5797C>T, p.(Arg1933Ter)<br>c.8584G>T, p.(Glu2862Ter)	Klinisch-Genetisch Centrum Nijmegen, Netherlands (2010)	NA
P71	*AQP5*<br>c.562C>T, p.(Arg188Cys)	Department of Clinical Genetics, Aarhus University Hospital, Denmark	Exome-sequencing-based *in-silico* panel containing 59 genes related to palmoplantar keratoderma
P19	*ABCA12*<br>c.1002_1004delAAC insT, p.(Thr335Alafs*5)<br>/ c.6263T>C, p.(Leu2088Pro)	Previously published[25]	
P31	*CARD14*<br>c.412G>A, p.(Glu138Lys)	Previously published[22]	
P32	*KRT1*<br>c.1424T>C, p.(Leu475Pro)	Previously published[21]	
P44	*KRT1*<br>c.673_702del30, p.His225_Phe234del	Previously published[21]	

© 2024 Gram SB et al, *JAMA Dermatology*.

eFigure 1
Examples of different clinical features in study subjects with palmoplantar keratoderma.

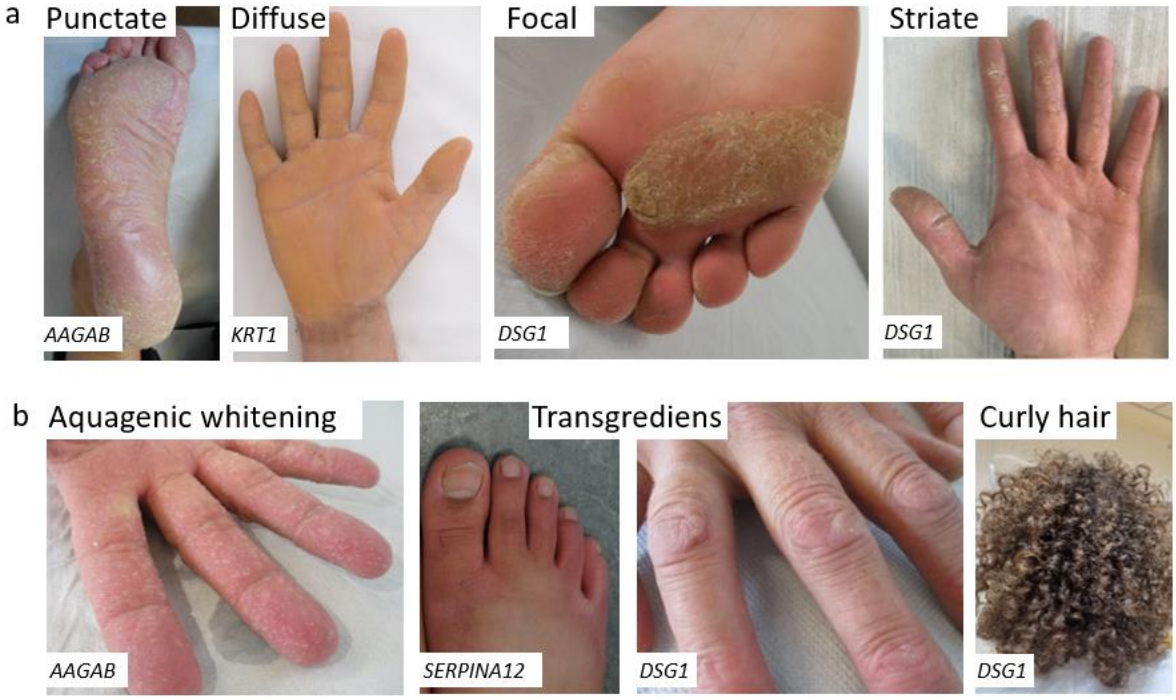

Clinical images of study subjects with palmoplantar keratoderma. Panel A. Illustrates the four different clinical subtypes based on the pattern of hyperkeratinisation. Panel B: Shows other clinical features i.e. aquagenic whitening, two examples of a transgredient pattern, and curly hair.

© 2024 Gram SB et al, *JAMA Dermatology*.

eFigure 2. Selected pedigrees of families included in the study

Family 8. *AAGAB,* c.370C>T, p.(Arg124Ter)

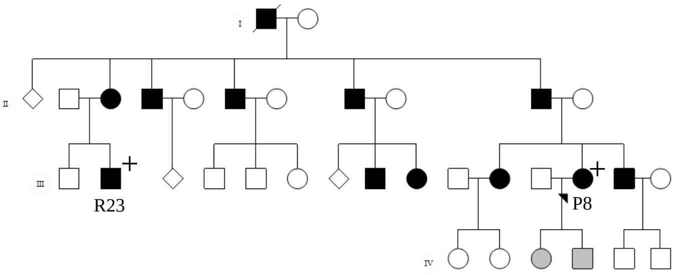

Family 4. *LORICRIN,* c.792dupC, p.(Ile265Hisfs*71)

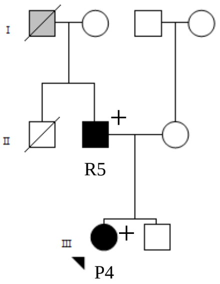

Family 21. *DSP,* c.2821C>T, p.(Arg941Ter)

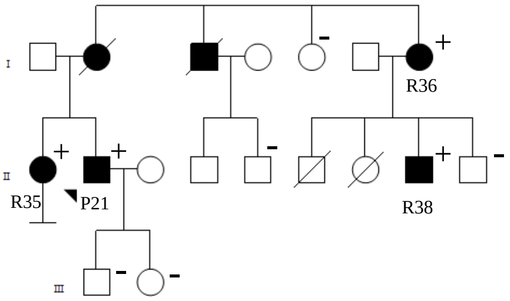

Family 60. *DSP, c.175dupA*, p.(Thr59Asnfs*34)

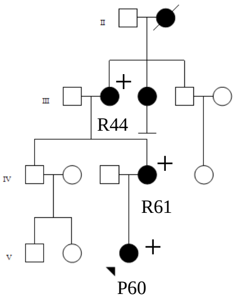

Family 46. *AQP5,* c.562C>T, p.(Arg188Cys)

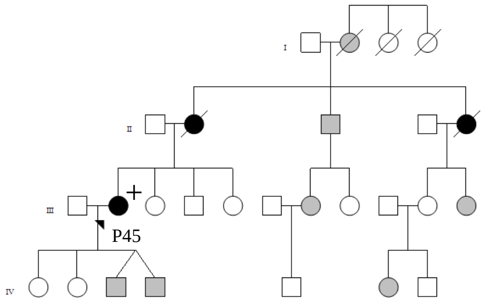

Family 73. *KRT1,* c.608A>G, p.(Gln203Arg)

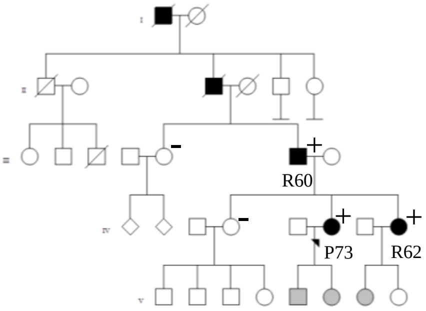

Family 50. *DSG1*, c.1947_1950delGAGA, p.(Arg650Ter)

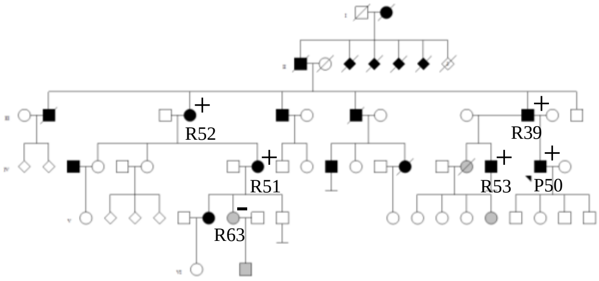

Family 26. *DSG1* c.2659C>T, p.(Arg887Ter)

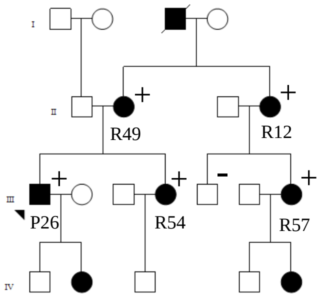

© 2024 Gram SB et al, *JAMA Dermatology*.

Patient ID: P=proband (also marked with at black arrowhead). R= relative. Blank indicates palmoplantar keratoderma, grey represents possible palmoplantar keratoderma. ‘+’ indicates individuals tested positive for the variant listed above the pedigree, ‘–‘ indicates individuals tested without the variant above the pedigree.

© 2024 Gram SB et al, *JAMA Dermatology*.

eFigure 3. Supporting evidence that the variant *ABCA12*, c.6263T>C, p.(Leu2088Pro) affects the protein function.

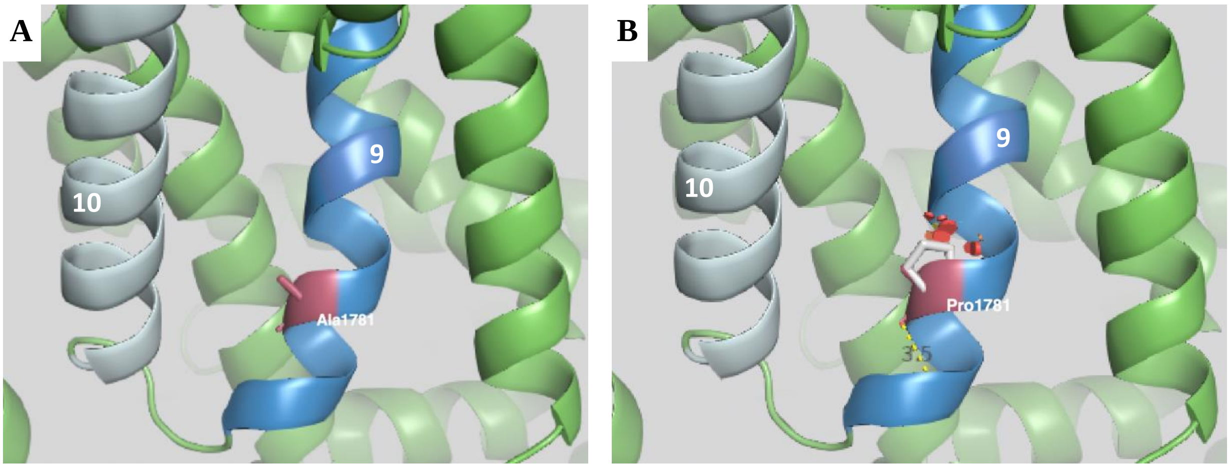

The figure shows two images of *ABCA4*, a paralogous protein to, *ABCA12*. In the alignment between *ABCA12* and *ABCA4*, leucine at position 2088 from *ABCA12* is substituted by alanine at position 1781 (red colour) in *ABCA4*. In panel A it is illustrated how Ala1781 is located in alpha-helix 9 (marine blue) immediately followed by a sharp loop turn connecting to alpha helix 10 (light blue) both of the transmembrane domain. In panel B it is illustrated how the introduction of proline at this position prevents the sharp turn (the distance to surrounding amino acids becomes too small), and there will be conformational overlap with Met1778 and Tyr1779 (illustrated by the red discs). The genetic variation in *ABCA4* p.Ala1781Glu is known to be disease-causing and result in Stargardt disease (PMID: 30060493). In *ABCA1* the paralog variant p.Ala1756Thr is known as a pathogenic genetic variant causing Tangier disease (PMID: 33066695). Further supporting data can be found in Table S3.

© 2024 Gram SB et al, *JAMA Dermatology*.

eFigure 4. Suggested genetic test strategy based on clinical subtype.

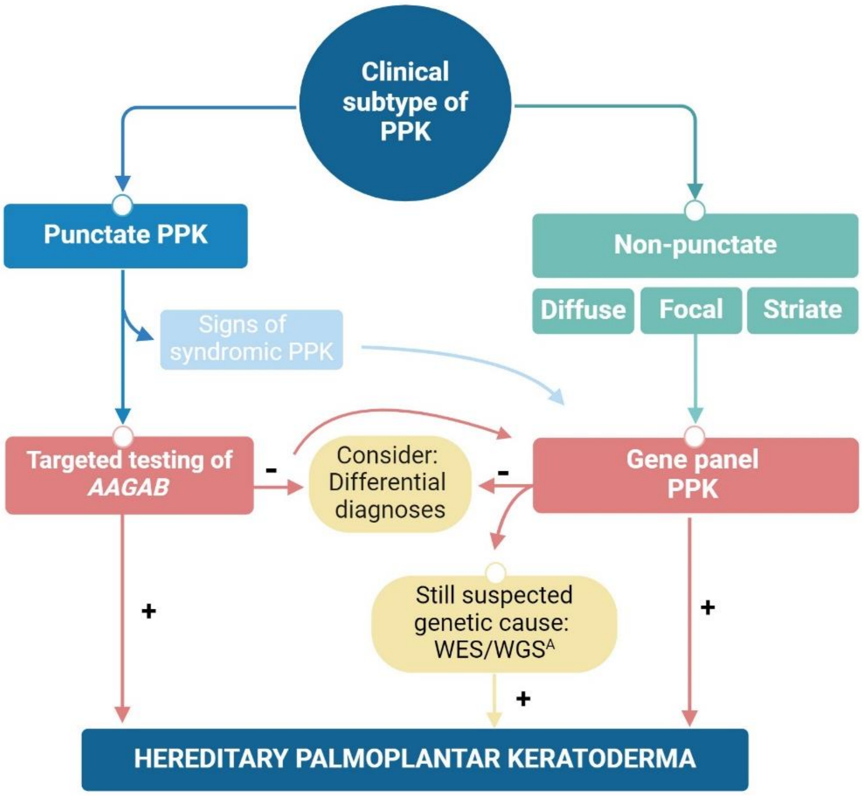

© 2024 Gram SB et al, *JAMA Dermatology*.

eTable 1. Clinical findings and familiar data on different genotypes identified in a cohort with palmoplantar keratoderma

Gene	Family	Proband			Family history of PPK	Affected (unaffected) relatives in the study	Condition associated with the gene (OMIM)	Other clinical findings
		ID	Sex/ age	Type				
AAGAB	35 cases / families	[A]		Punctate	Y (n=32) N (n=2) NA (n=1)		Palmoplantar keratoderma, punctate type 1 (#148600)	–
	Family 34	P34	M/70	Punctate	Y	–		–
	Family 42	P42	F/59	Punctate	Y	1		–
	Family 52	P52	F/54	Punctate	Y			–
	Family 58	P58	M/55	Punctate	Y	–		–
DSG1	Family 2	P2	M/42	Diffuse	Y	2 ([B])	Palmoplantar keratoderma I, striate, focal, or diffuse (OMIM#148700)	The pattern of hyperkeratinisation exhibited considerable diversity among study participants with *DSG1* variants, showing a combined pattern of diffuse, focal, and striate PPK. This diversity was evident both on an individual patient level and inter- and intrafamilial.
	Family 23	P23	M/28	Striate	N	–		
	Family 26	P26	M/39	Diffuse	Y	4 (1)		
	Family 41	P26	M/35	Striate	Y	–		
	Family 45	P45	F/47	Diffuse[C]	Y	4		
	Family 48	P48	M/27	Focal	Y	-		
	Family 50	P50	M/38	Diffuse[C]	Y	4 ([B])		
	Family 68	P68	F/25	Striate	Y	1		
KRT9	Family 30	P30	F/34	Diffuse	Y	–	Palmoplantar keratoderma, epidermolytic (OMIM#144200)	Extensive keratoderma on palms and soles
	Family 43	P43	F/26	Diffuse	Y	–		Extensive keratoderma on palms and soles. Hypercurved nails.
AQP5	Family 46	P46	F/55	Diffuse	Y	–	Palmoplantar keratoderma, Bothnian type (OMIM#600231)	–
	Family 71	P71	F/19	Diffuse	Y	1		–
KRT16	Family 13	P13	M/31	Diffuse[C]	N	– (2)	Pachyonychia congenita 1 (OMIM#167200) + palmoplantar keratoderma, nonepidermolytic, focal (OMIM#613000)	Only marginally noticeable signs of keratoderma on the fingertips. Four thickened toenails. The patient reported reduced amount of sweat in general (not limited to hands and feet), but did not report any signs of oral leukokeratosis, ear wax or ear pain, hoarseness or history of natal teeth or nursing difficulties.

© 2024 Gram SB et al, *JAMA Dermatology*.

eTable 1, continued: Clinical findings and familiar data on different genotypes identified in a cohort with palmoplantar keratoderma

KRT1	Family 73	P73	F/38	Diffuse	Y	3 (2)	Palmoplantar keratoderma, epidermolytic[D], 2 (OMIM#620411)	−
KRT1	Family 32	P32	M/32	Diffuse	N	− (2)	Epidermolytic hyperkeratosis 1 (OMIM#113800)	Epidermolytic hyperkeratosis with ichthyosis
	Family 44	P44	F/45	Diffuse	Y	− (2)		Epidermolytic hyperkeratosis with ichthyosis
LORICRIN	Family 4	P4	F/24	Diffuse	Y	1	Vohwinkel syndrome with ichthyosis (OMIM #604117)	Proband had yellowish keratoderma, minor signs of pseudoainhum on fifth fingers' distal phalanx, mild scaling of the skin consistent with diffuse ichthyosis. The father had pronounced dryness and scaling on the palms and mild scaling skin on other body areas.
SERPINA12	Family 64	P4	F/21	Diffuse	Y	-	-	Diffuse, erythematous, transgredient keratoderma
CARD14	Family 31	P31	F/70	Focal	N	−	Pityriasis rubra pilaris (OMIM#173200)	Pityriasis rubra pilaris
DST	Family 59	P59	F/27	Focal	N	−	Epidermolysis bullosa simplex 3 (OMIM#615425)	Less classical clinical presentation with both blisters and keratoderma consistent with the genetic diagnosis
COL7A1	Family 63	P63	M/41	Diffuse[C]	N	−	Epidermolysis bullosa dystrophica (OMIM#226600)	Recessive dystrophic epidermolysis bullosa, intermediate
ABCA12	Family 19	P19	F/75	Diffuse	N	−	Ichthyosis, congenital, autosomal recessive 4A (OMIM#601277)	Congenital ichthyosis
DSP	Family 21	P21	M/67	Diffuse	Y	3 (5)	Dilated cardiomyopathy with woolly hair, keratoderma, and tooth agenesis (OMIM #615821) and Keratosis palmo-plantaris striata II (OMIM#612908)	Cardiac examination of proband and sister (F/65y) was normal. The mother's sister (84y) was reported to have unspecific cardiac findings which may be indicative of DSP-related disease. Her son had a history of two episodes of perimyocarditis. His sister died suddenly in her early twenties. No information on either PPK or cause of death was available.
	Family 60	P60	F/18	Focal	Y	2		Proband only affected on soles. Relatives had mild plantar keratosis, but were barely affected on palms. Cardiac examinations were normal for the affected family members.

Y: yes. N: A) P3,P5,P8-P12,15,P18,P20,P22,P24-25,P28-29,P33,P35-39, P47,P49, P51,P53-56. B) In both family 2 and family 50, one relative was initially included in the study with self-reported PPK. In both cases, clinical images showed only mild signs of plantar keratodermas. Genetic testing did not reveal the variant identified in the family. It was not possible to perform additional clinical examinations and/or obtain new blood samples. C) In the marked cases, the classifications of the clinical subtypes proved to be challenging, leading to discussion regarding the different cases as follows: P45=diffuse or focal. P50=diffuse or focal. *KRT16*=diffuse or focal. *COL7A1* = mainly diffuse, but with a few punctate elements. D) A biopsy performed in the affected sister (R62) was consisted with epidermolytic palmoplantar keratoderma.

© 2024 Gram SB et al, *JAMA Dermatology*.

eTable 2. Demographics and clinical presentation of 117 study subjects (probands and relatives) with molecular genetic confirmed palmoplantar keratoderma. Detailed informatic [illegible] to each genotype is listed.

	AAGAB	*DSG1*	*DSP*	*KRT1*	*AQP5*	*LORICRIN*	*KRT9*	*KRT16*	*CARD14*	*ABCA12*	*DST*	*COL7A1*	*SERPINA12*	Stud[illegible] gen[illegible]
**Number of study subjects**	**n=69**	**n=23**	**n=7**	**n=5**	**n=3**	**n=2**	**n=2**	**n=1**	**n=1**	**n=1**	**n=1**	**n=1**	**n=1**	
Probands	39	8	2	3	2	1	2	1	1	1	1	1	1	
Relatives (from no. of families)	30 (18)	15 (5)	5 (2)	2 (1)	1 (1)	1 (1)	0	0	0	0	0	0	0	
**Demographics**														
Sex (M/F)	25/44	10/13	2/5	2/3	1/2	1/1	0/2	1/0	0/1	0/1	0/1	1/0	0/1	
Age at inclusion	56.1 (19-92)	46.3 (18-87)	55.1 (18-84)	43.2 (30-71)	40.3 (19-55)	40 (24-56)	30 (26-34)	31	70	75	27	41	21	[illegible]
**Type**														
Punctate	69/69													
Non-punctate		22/23	7/7	5/5	3/3	2/2	2/2	1/1	1/1	1/1	1/1	1/1	1/1	
**Palms and soles**														
Palms AND soles	67/69	21/21	6/7	5/5	3/3	2/2	2/2	1/1	1/1	1/1			1/1	
Only palms														
Only soles	2/69		1/7								1/1	1/1		
**Age of onset** *years, median (range)*	18 (4,5-47)	5.5 (0-14)	7.5 (4-19)	2 (0-14,5)	10 (4-10)	11 (6-16)	0	5-6	NA	NA	18	36	0	
**Age of onset by period**														
At birth				1/5 (20)										
< 1 year		2/23 (9)		1/5 (20)			2/2 (100)						1/1 (100)	
1-9 years	4/69 (6)	16/23 (70)	3/7 (43)	2/5 (40)				1/1 (100)						[illegible]
10-19 years	32/69 (46)	5/23 (22)	3/7 (48)	1/5 (20)	1/3 (33)	1/2 (50)					1/1 (100)			[illegible]
> 19 years	29/69 (42)				2/3 (67)	1/2 (50)						1/1 (100)		[illegible]
Other	4/69 (6)		1/7 (14)						1/1 (100)	1/1 (100)				
**Disease progression with age**														
Stable	14/69 (20)	10/23 (43)	1/7 (14)	4/5 (80)	2/3 (67)		1/2 (50)		1/1 (100)		1/1 (100)		1/1 (100)	[illegible]
Improvement		8/23 (35)	3/7 (46)				1/2 (50)							[illegible]
Worsening	54/69 (78)	5/23 (43)	1/7 (14)	1/5 (20)	1/3 (33)	2/2 (100)								[illegible]
Other	1/69 (1)		2/7 (29)					1/1 (100)		1/1 (100)				
**Associated symptoms**														
Pain/sore	46/69 (67)	21/23 (91)	5/7 (71)	3/5 (60)	2/3 (67)	0/2 (0)	2/2 (100)	1/1 (100)	1/1 (100)	0/1 (0)	0/1 (0)	1/1 (100)	0/1 (0)	[illegible]
Sweat	28/64 (44)	11/22 (50)	0/7 (0)	2/5 (40)	3/3 (100)	½ (50)	0/1 (0)	1/1 (100)	0/1 (0)	0/1 (0)	0/0 (0)	0/1 (0)	1/1 (100)	[illegible]
Odour	30/66 (46)	14/23 (61)	1/7 (14)	2/5 (40)	2/3 (100)	½ (50)	½ (50)	0/1 (0)	0/1 (0)	0/1 (0)	1/1 (100)	0/1 (0)	1/1 (100)	[illegible]
Fungal infections	9/65 (14)	9/22 (41)	2/7 (29)	1/4 (35)	2/3 (100)	0/2 (0)	0/1 (50)	1/1 (100)	1/1 (100)	0/1 (0)	0/1 (0)	0/1 (0)	0/1 (0)	[illegible]
Aquagenic whitening	59/67 (88)	16/21 (76)	2/5 (40)	5/5 (100)	3/3 (100)	2/2 (100)	2/2 (100)	1/1 (100)	0/1 (0)	0/0 (0)	0/1 (0)	0/1 (0)	0/1 (0)	[illegible]

© 2024 Gram SB et al, *JAMA Dermatology*.

eTable 3 Supplementary information on the genetic variants identified in the cohort including information on reference sequences, zygosity, gnomAD, software and ACMG-criteria and interpretation.

Proband_ID	Gene	Nucleotide change	Amino acid change	Exon	Reference sequence (GrCh37)	Zygosity	Inheritance	gnomAD v. 2.1.1	Software (SIFT:Polyphen2:CADD)	ACMG criteria
P2	*DSG1*	c.1421delC	p.(Thr474Ilefs*88)	11 of 15	NM_001942.3	Het	AD (maternal)	0	_:_:27.3	PVS1, PM2
P23	*DSG1*	c.1005+1G>T	p.?	intron 8	NM_001942.3	Het	De novo	0	_:_:33 [a]	PVS1, PM2, PM6, PP3
P26	*DSG1*	c.2659C>T	p.(Arg887Ter)	15 of 15	NM_001942.4	Het	AD (maternal)	0	_:_:39	PVS1, PM2, PP1
P41	*DSG1*	c.76C>T	p.(Arg26*)	2 of 15	NM_001942.4	Het	AD (maternel)	0	_:_:34	PVS1, PS1, PM2
P45	*DSG1*	c.1199C>G	p.(Ser400Ter)	9 of 15	NM_001942.4	Het	AD (paternal)	0	_:_ :32	PVS1, PM2, PP1
P48	*DSG1*	c.(372_373)_(1005+1006)del	p.?	5 to 8	NM_001942.4	Het	AD (paternal)	0	_:_ :NA	PVS1, PM2
P50	*DSG1*	c.1947_1950delGAGA	p.(Arg650Ter)	14 of 15	NM_001942.4	Het	AD (paternal)	0	_: _ :38	PVS1, PM2
F68	*DSG1*	c.1265+1G>A	p.?	intron 9	NM_001942.4	Het	AD (maternal)	0	_:_ :34	PVS1, PM2
P21	*DSP*	c.2821C>T	p.(Arg941Ter)	20 of 24	NM_004415.3	Het	AD (maternal)	0	_:_:43	PVS1, PM2, PP1
F60	*DSP*	c.175dupA	p.(Thr59Asnfs*34)	2 of 24	NM_004415.4	Het	AD (maternal)	0	_: _:40	PVS1, PM2
P30	*KRT9*	c.487C>T	p.(Arg163Trp)	1 of 8	NM_000226.3	Het	AD (maternal)	0	D:PD:23	PS1, PS3, PM1, PM2, PP3
P43	*KRT9*	c.487C>T	p.(Arg163Trp)	1 of 8	NM_000226.3	Het	AD (paternal)	0	D:PD:23	PS1, PS3, PM1, PM2, PP3
P46	*AQP5*	c.562C>T	p.(Arg188Cys)	3 of 8	NM_001651.4	Het	AD(maternal)	19/282800	D:PD:25.3	PS1, PS3, PP3
F71	*AQP5*	c.562C>T	p.(Arg188Cys)	3 of 8	NM_001651.4	Het	AD (paternal)	19/282800	D:PD:25.3	PS1, PS3, PP3
P4	*LORICRIN*	c.792dupC	p.(Ile265Hisfs*71)	2 of 2	NM_000427.3	Het	AD (paternal)	0	_:_:12.59	PVS1, PM2, PM4
P13	*KRT16*	c.379C>T	p.(Arg127Cys)	1 of 8	NM_005557.3	Het	De novo	0	D:B:26.1	PS1, PM1, PM2, PM6
P32	*KRT1*	c.1424T>C	p.(Leu475Pro)	7 of 9	NM_006121.3	Het	De novo	0	D:PD:26.2	PS1, PM2, PM6, PP3
P44	*KRT1*	c.673_702del30	p.His225_Phe234del	2 of 9	NM_006121.3	Het	De novo	0	_: _:_	PVS1, PM2, PM4, PM6
P73	*KRT1*	c.608A>G	p.(Gln203Arg)	2 of 9	NM_006121.4	Het	AD (paternal)	0	D:PD:27	PM1, PM2, PP1, PP3
P31	*CARD14*	c.412G>A	p.(Glu138Lys)	4 of 21	NM_024110.4	Het	NA	0	D:PD:24.9	PS1, PM2, PP3
P59	*DST*	c.7544dupA	p.(Gln2516Alafs*6)	24 of24	NM_001723.5	Homo	AR	2/282610	_: _:19.1	PVS1, PM2, PM3
P64	*SERPINA12*	c.594delC	p.Gly199Alafs*3	2 of 5	NM_001382267.1	Homo	AR	0	_: _:17.6	PVS1, PM2
P63	COL7A1	c.5797C>T /	p.(Arg1933Ter) /	70 of 118	NM_000094.2	Compound het.	AR	0	_: _: 35	PVS1, PS1, PM2, PM3
		c.8584G>T	p.(Glu2862Ter)	116 of 118				0	_: _:41	PVS1, PS1, PM2, PM3.
P19	*ABCA12*	c.1002_1004delAACinsT /	p.(Thr335Alafs*5) /	9 of 53	NM_173076.2	Phase unknown	AR	0	_: _:_	PVS1, PM2
		c.6263T>C	p.(Leu2088Pro)	43 of 53				2/251290	D:PB:29.3	PM2, PP3.
P34	*AAGAB*	c.2T>A	p.Met1?	1 of 10	NM_024666.5	Het	AD (paternal)	0	D:PD:24.2	PVS1, PM2, PM2
P42	*AAGAB*	(chr15: 67534480_67579632)del	p.?	1 of 10	NC_000015.10 (NM_024666.5)	Het	AD (NA)	0	_: _:_	PVS1, PM2
P52	*AAGAB*	(chr15: 67534480_67579632)del	p.?	1 of 10	NC_000015.10 (NM_024666.5)	Het	AD (NA)	0	_: _:_	PVS1, PM2
P58	*AAGAB*	(c.1-6607)_(c.74-95)del	p.?	1 of 10	NM_024666.5	Het	AD (NA)	0	_: _:_	PVS1, PM2
P3, P5, P8-P12, P15, P18, P20, P22, P24-25, P28-P29, P33, P35-39, P47, P49, P51, P53-P56, P61, P65-P66, P67, P70, P72, P75	*AAGAB*	c.370C>T	p.(Arg124Ter)	4 of 10	NM_024666.4 / NM_024666.5	Het	AD (maternel; n=11) AD (paternel; n=19)	7/279006	_:_:36	PVS1, PS1, PS4

AD: autosomal dominant. AR: autosomal recessive. D=damaging. PD=probably damaging. B=benign. a) SpliceSiteFinder-like/MaxEntScan/NNSPLICE/GeneSplicer all predicted effect on splicing . Het = heterozygous. Homo = homozygous.

© 2024 Gram SB et al, *JAMA Dermatology*.

eTable 4. Variants of unknown significance identified in the 76 probands with PPK

Proband_ID	Gene	Nucleotide change	Amino acid change	Zygocity	Reference sequence (GrCh37)	Variant found in a case with an alternate molecular basis for disease
P2	*DSP*	c.273+5G>A	p.?	Heterozygous	NM_004415.3	Y (*DSG1*)
P5	*COL14A1*	c.3705G>A	p.(Met1235Ile)	Heterozygous	NM_021110.3	Y (*AAGAB*)
P6	*WNT10A*	c.810C>A	p.(Ser270Arg)	Heterozygous	NM_025216.2	N
P9	*KRT9*	c.1011C>A	p.(Asn337Lys)	Heterozygous	NM_000226.3	Y (*AAGAB*)
P12	*ATP2A2*	c.1120G>A,	p.(Gly374Ser)	Heterozygous	NM_001681.3	Y (*AAGAB*)
P21	*DSP*	c.505G>T	p.Gly169Cys	Heterozygous	NM_004415.3	Y (*DSP*)
P41	*JUP*	c.560C>T	p.Ala187Val	Heterozygous	NM_002230.4	Y (*DSG1*)
P42	*COL20A1*	c.2345G>A	p.(Gly782Glu)	Heterozygous	NM_020882.2	Y (*AAGAB*)
P45	*JUP*	c.427G>A	p.Ala143Thr	Heterozygous	NM_002230.4	Y (*DSG1*)
P48	*KRT17*	c.850C>T	p.(Arg284Cys)	Heterozygous	NM_000422.3	Y (*DSG1*)
P64	*DSP*	c.4901G>A,	p.(Arg1634Gln)	Heterozygous	NM_004415.4	N
P68	*SERPINB7*	c.1136G>A	p.(Cys379Tyr)	Heterozygous	NM_003784.4	Y (*DSG1*)

© 2024 Gram SB et al, *JAMA Dermatology*.

eTable 5. Clinical characteristics of the 13 study participants without a genetic diagnosis

Type PPK		Proband ID		Age of onset	Palms and/ or soles	Transgrediens	Family history (suspected inheritance)	No. of additional affected family members
Punctate	Family 7	P7	M/56	47	Both	NA	Yes (AD)	3
	Family 27	P27	F/32	10	Both	NA	No	-
	Family 40	P40	F/73	NA	Soles	NA	Maybe	(1)
	Family 76	P76	F/24	21	Both	No	No	-
Diffuse	Family 1	P1	F/19	0	Both	Yes (erythematous)	Yes (AD)	2
	Family 16	P16	F/33	Childhood	Both	Yes (erythematous)	Yes (AD)	6
	Family 62	P62	F/62	NA	Both	Yes (erythematous)	Yes (AD)	1
	Family 69	P69	M/25	0	Both	Yes (erythematous)	Yes (AD)	13
	Family 6	P6	F/19	11-12	Both	NA	Yes (AD)	3
	Family 14	P14	M/49	44	Soles	NA	Yes (AD)	2
	Family 17	P17	F/63	10-11	Both	NA	Yes (AD)	4
	Family 57	P57	F/45	30	Both	Yes	No	-
	Family 74	P74	M/53	20	Both	No	Perhaps	-

© 2024 Gram SB et al, *JAMA Dermatology*.
