# Supplementary material for: Clinical and Genetic Findings in Patients With Palmoplantar Keratoderma
Source: JAMA Dermatol. 2024 Dec 4;161(2):157–66. doi: 10.1001/jamadermatol.2024.4824 (PMC11618570; doi:10.1001/jamadermatol.2024.4824)
Supplement: Supplement 2. — Data Sharing Statement [file jamadermatol-e244824-s002.pdf]

Data Sharing Statement

Gram. Clinical and Genetic Findings in Patients With Palmoplantar Keratoderma. *JAMA Dermatol*. Published December 04, 2024. doi:10.1001/jamadermatol.2024.4824

Data
Data available: Yes
Data types: Other (please specify)
Additional Information: The data that support the findings of this study are available from the corresponding author, [SBG], upon reasonable request.
How to access data: Stine.Bjorn.gram@rsyd.dk
When available: With publication

Supporting Documents
Document types: None

Additional Information
Who can access the data: Depends of which data is requested.
Types of analyses: Depends of which data is requested.
Mechanisms of data availability: Depends of which data is requested.
